# Genomic insights into ESBL-producing *Escherichia coli* isolated from non-human primates in the Peruvian Amazon

**DOI:** 10.3389/fvets.2023.1340428

**Published:** 2024-01-16

**Authors:** Jhonathan Bazalar-Gonzales, Thalía Silvestre-Espejo, Carmen Rodríguez Cueva, Dennis Carhuaricra Huamán, Yennifer Ignación León, Luis Luna Espinoza, Raúl Rosadio Alcántara, Lenin Maturrano Hernández

**Affiliations:** ^1^Research Group in Biotechnology Applied to Animal Health, Production and Conservation (SANIGEN), Laboratory of Biology and Molecular Genetics, Faculty of Veterinary Medicine, Universidad Nacional Mayor de San Marcos, Lima, Peru; ^2^Asociación Equipo Primatológico del Perú (EPP), Iquitos, Peru; ^3^Programa de Pós-Graduação Interunidades em Bioinformática, Instituto de Matemática e Estatística, Universidade de São Paulo, São Paulo, Brazil

**Keywords:** antimicrobial resistance, extended-spectrum beta-lactamase, *Escherichia coli*, non-human primates, Peruvian Amazon, molecular epidemiology, multilocus sequence typing, phylogenomic

## Abstract

**Introduction:**

Extended-spectrum beta-lactamase (ESBL)-producing *Enterobacteriaceae* are on the WHO priority pathogens list because they are associated with high mortality, health-care burden, and antimicrobial resistance (AMR), a serious problem that threatens global public health and should be addressed through the One Health approach. Non-human primates (NHP) have a high risk of acquiring these antibiotic-resistant bacteria due to their close phylogenetic relationship with humans and increased anthropogenic activities in their natural environments. This study aimed to detect and analyze the genomes of ESBL-producing *Escherichia coli* (ESBL-producing *E. coli*) in NHP from the Peruvian Amazon.

**Materials and methods:**

We collected a total of 119 fecal samples from semi-captive *Saguinus labiatus*, *Saguinus mystax*, and *Saimiri boliviensis*, and captive *Ateles chamek*, *Cebus unicolor*, *Lagothrix lagothricha*, and *Sapajus apella* in the Loreto and Ucayali regions, respectively. Subsequently, we isolated and identified *E. coli* strains by microbiological methods, detected ESBL-producing *E. coli* through antimicrobial susceptibility tests following CLSI guidelines, and analyzed their genomes using previously described genomic methods.

**Results:**

We detected that 7.07% (7/99) of *E. coli* strains: 5.45% (3/55) from Loreto and 9.09% (4/44) from Ucayali, expressed ESBL phenotype. Genomic analysis revealed the presence of high-risk pandemic clones, such as ST10 and ST117, carrying a broad resistome to relevant antibiotics, including three *bla*_CTX-M_ variants: *bla*_CTX-M-15_, *bla*_CTX-M-55_, and *bla*_CTX-M-65_. Phylogenomic analysis confirmed the clonal relatedness of high-risk lineages circulating at the human-NHP interface. Additionally, two ESBL-producing *E. coli* strains were identified as EPEC (*eae*) and ExPEC according to their virulence profiles, and one more presented a hypermucoviscous phenotype.

**Discussion:**

We report the detection and genomic analysis of seven ESBL-producing *E. coli* strains carrying broad resistome and virulence factors in NHP from two regions of the Peruvian Amazon. Some of these strains are closely related to high-risk pandemic lineages previously reported in humans and domestic animals, highlighting the negative impact of anthropogenic activities on Amazonian wildlife. To our knowledge, this is the first documentation of ESBL-producing *E. coli* in NHP from the Amazon, underscoring the importance of adopting the One Health approach to AMR surveillance and minimizing the potential transmission risk of antibiotic-resistant bacteria at the human-NHP interface.

## 1 Introduction

The emergence and spread of antimicrobial resistance (AMR) represent a major threat to global public health, with low and middle-income countries (LMICs) disproportionately affected (1, 2). The AMR challenge in LMICs extended beyond medical treatments for the human population, as a high prevalence of antibiotic-resistant bacteria is observed in intensive livestock production due to the unregulated use of antibiotics as growth promoters, despite being prohibited in many countries, along with inadequate veterinary treatments (3). Antibiotic-resistant bacteria from healthcare and farm settings may run off into natural environments, eventually reaching wildlife. Consequently, there is an increased need to promote a One Health approach to comprehend and address the emergence and evolution of AMR (4, 5).

According to the World Health Organization (WHO), extended-spectrum beta-lactamase (ESBL) producing *Enterobacteriaceae* rank among the most critical antibiotic-resistant bacteria (6). These bacteria produce enzymes that hydrolyze beta-lactam antibiotics, including third- and fourth generation cephalosporins. The *bla*_CTX-M_ gene family, the most widespread and clinically relevant ESBL enzyme, can be easily transmitted via plasmids, facilitating rapid spread and dissemination (7). There is evidence demonstrating the presence of ESBL-producing *Escherichia coli* (ESBL-producing *E. coli*) in the gut microbiomes of domestic and wild animals, with rapid dissemination observed in wildlife around the world (8–10).

Wild animals, including non-human primates (NHP), are generally not exposed to antibiotics; however, they can acquire antibiotic-resistant bacteria, such as ESBL-producing *E. coli*, through foraging and drinking in natural environments contaminated by anthropogenic sources (11). Moreover, forest fragmentation resulting from anthropogenic activities such as agriculture, livestock, and forestry, along with changes in the lifestyle of free-living NHP transitioning to captive and semi-captive conditions, can increase the interaction between humans and NHP and enhance the spread of these antibiotic-resistant bacteria (12).

Peru boasts an wide biodiversity of neotropical fauna residing in different regions of the Peruvian Amazon (13), including various NHP classified into 55 taxa comprising species and subspecies. Around 30% of these are designated as “threatened with extinction,” five as “near threatened” and five more as “data deficient,” according to the International Union for the Conservation of Nature (IUCN) (14). However, few studies have investigated AMR in Peruvian Amazon wildlife, and only one has reported antibiotic-resistant *Enterobacteriaceae* from semi-captive NHP of *Ateles*, *Callicebus*, and *Lagothrix* genus, with *E. coli* the most prevalent enterobacteria (15), but not detected their antimicrobial resistance genes (ARGs).

This study aimed to detect the presence of ESBL-producing *E. coli* strains in captive and semi-captive NHP from two regions of the Peruvian Amazon and analyze their genomes. We performed the isolation and identification of *E. coli* strains using microbiological methods, the phenotypic detection of ESBL-producing *E. coli* through antimicrobial susceptibility tests according to the Clinical and Laboratory Standard Institute (CLSI), identification of ARGs within the resistome, and the analysis of virulence genes using genomic approaches. Additionally, we conducted a phylogenomic analysis of high-risk lineages.

## 2 Materials and methods

### 2.1 Ethics approval statement

This study obtained authorization for scientific research purposes on wildlife outside Protected Natural Areas from the National Forestry and Wildlife Service of Peru (General Directorate Resolution No. RD-000031-2022-MIDAGRI-SERFOR-DGGSPFFS-DGSPFS). All procedures have undergone review and approval by the Ethics and Animal Welfare Committee of the Faculty of Veterinary Medicine of the Universidad Nacional Mayor de San Marcos (Authorization No. 2022-10).

### 2.2 Fecal sampling collection

In August 2022, we collected a total of 119 fecal samples from semi-captive and captive NHP in two regions of the Peruvian Amazon. Of these, 69 samples belonged to semi-captive NHP specimens, including *Saimiri boliviensis* (*n* = 26), *Saguinus labiatus* (*n* = 21), and *Saguinus mystax* (*n* = 23), that cohabit with humans in rainforests near urban–rural areas on Iquitos, Muyuy, and Padre islands, respectively, in the Loreto region of the northern Peruvian Amazon. Additionally, 50 samples were obtained from captive NHP specimens, including *Ateles chamek* (*n* = 14), *Cebus unicolor* (*n* = 11), *Lagothrix lagothricha* (*n* = 14), and *Sapajus apella* (*n* = 11), housed in a Rescue Center and a Zoo that are frequently visited by many local and foreign people, in the Ucayali region of the eastern Peruvian Amazon.

Briefly, we collected between 5 and 10 mg of recently excreted feces using a sterile disposable palette, avoiding the parts that had direct contact with the ground and other surfaces to minimize the risk of sample contamination. The samples were placed inside sterile fecal containers, labeled according to their characteristics (species, sampling region, and living conditions), packed in a cooler container with controlled refrigeration temperature (4°C), and sent to the Laboratory of the Biology and Molecular Genetics of the Faculty of Veterinary Medicine at the Universidad Nacional Mayor de San Marcos for processing using microbiological and molecular methods.

### 2.3 Microbiological isolation and identification of *Escherichia coli* strains

The fecal samples were diluted in sterile tubes with isotonic saline solution (0.9% NaCl) in a 1:1 ratio and gently mixed through vortexing. Subsequently, the samples were streaked onto MacConkey agar (Sigma-Aldrich, Germany) and incubated in an Incucell-ecoline incubator model (MMM Group, Germany) at 37°C for 24 h under aerobic conditions. On the following day, all lactose-positive colonies displaying a brick-red to rosaceous color surrounded by a zone of precipitated bile were selected and streaked onto Eosin-Methylene Blue (EMB) agar, followed by incubation under the same conditions. Colonies exhibiting a metallic green luster were then chosen and confirmed through IMViC (Indole, Methyl Red, Voges-Proskauer and Citrate test) biochemical tests (Merck, Germany). Additionally, a “String” test was also conducted on all strains with a mucoid appearance using sterile microbiological loops. This test involved inoculating our strains onto blood agar (Merck, Germany) with 5% sheep blood and incubating them at 37°C overnight, as previously described (16).

### 2.4 Antimicrobial susceptibility testing of *Escherichia coli* strains

Antimicrobial susceptibility testing was conducted using the Kirby-Bauer disk diffusion method, following the CLSI recommendations (17). In brief, *E. coli* strains were diluted in sterile tubes with distilled water to a turbidity of 0.5 on the McFarland standard scale. They were then transferred with sterile swabs onto Petri dishes containing Mueller-Hinton (MH) agar (Millipore, Germany). To determine the ESBL phenotype, a screening test was conducted using ceftazidime (30 μg), cefotaxime (30 μg), ceftriaxone (30 μg), and aztreonam (30 μg) antibiotic disks (Oxoid, United Kingdom). The plates were incubated for 18 h at 37°C under aerobic conditions, and the results were interpreted according to CLSI guidelines.

The following day, a confirmation test based on the Jarlier Method was conducted on all suspected strains. This involved placing an amoxicillin/clavulanic acid (20/10 μg) antibiotic disk in the center of a Petri dish with MH agar previously inoculated, surrounded by the antibiotic disks tested earlier. The dishes were then incubated under the same conditions, and strains that formed a distorted inhibition halo were considered ESBL-positive (18).

Additionally, the resistance profile phenotype was evaluated using oxytetracycline (30 μg), nitrofurantoin (300 μg), levofloxacin (5 μg), gentamicin (10 μg), trimethoprim/sulfamethoxazole (1.25/23.75 μg), and chloramphenicol (30 μg) antibiotics disks. Multidrug-resistant (MDR) strains were identified as those showing resistance to three or more classes of the antibiotics tested, following the definition provided by Magiorakos et al. (19).

### 2.5 Whole genome sequencing and assembly of ESBL-producing *Escherichia coli* strains

Total genomic DNA from all ESBL-producing *E. coli* strains was extracted, and paired-end libraries were sequenced on the Illumina MiSeq platform (Illumina, San Diego, CA, United States) using kit v3 (600-cycle) with 2 × 250 bp reads. To ensure data quality, the obtained FastQ files were trimmed using Trimmomatic (20), and quality assessment was conducted using FastQC (21). The cleaned reads were then submitted to the Enterobase database (22) for further analysis. Assembly, Multilocus sequence type (MLST), Clermont type, Hierarchical Clustering of core genome MLST (HierCC) clusters, and serotype statistics were retrieved from the Enterobase platform. The seven ESBL strains sequenced in this study have been deposited under the BioProject PRJNA992559 of the National Center for Biotechnology Information (NCBI).

### 2.6 Identification of sequence of interest: ARGs, virulence factors, capsule loci, and plasmids

To identify ARGs, we employed the AMRFinderPlus database (23) within the abriTAMR v 1.0.14 pipeline, applying a 90% identity and 90% coverage cutoff (24). Plasmid replicons, virulence genes, and *Klebsiella* capsule synthesis loci (K-locus) were identified from whole-genome data using the PlasmidFinder database (25), Virulence Factor Database (26) and Kaptive 2.0.7 (27), respectively. Additionally, gene cluster comparison figures were generated using Clinker v 0.0.27 (28).

### 2.7 Phylogenetic analysis

The phylogenomic analysis of the *E. coli* genomes obtained in this study and of the genetically related sequences deposited in Enterobase was conducted according to the HierCC HC50 or MLST scheme. Core alignment was performed using Snippy v 4.6.0.^1^ Gubbins (29) was employed to detect and eliminate recombinant regions. Maximum likelihood phylogenetic reconstruction was carried out using IQ-TREE v 2.0 (30) with GTR + G + I model and 1,000 bootstrap replicates. The resulting tree was visualized using the ggtree package (31) in the R environment v4.3 (32). Additionally, the pairwise SNP distance between genomes, derived from the free-recombination core genome alignment was calculated using SNP-dists v0.6 (see text footnote 1).

## 3 Results

### 3.1 Detection and resistome characterization of ESBL-producing *Escherichia coli* strains

**Figure 1:**
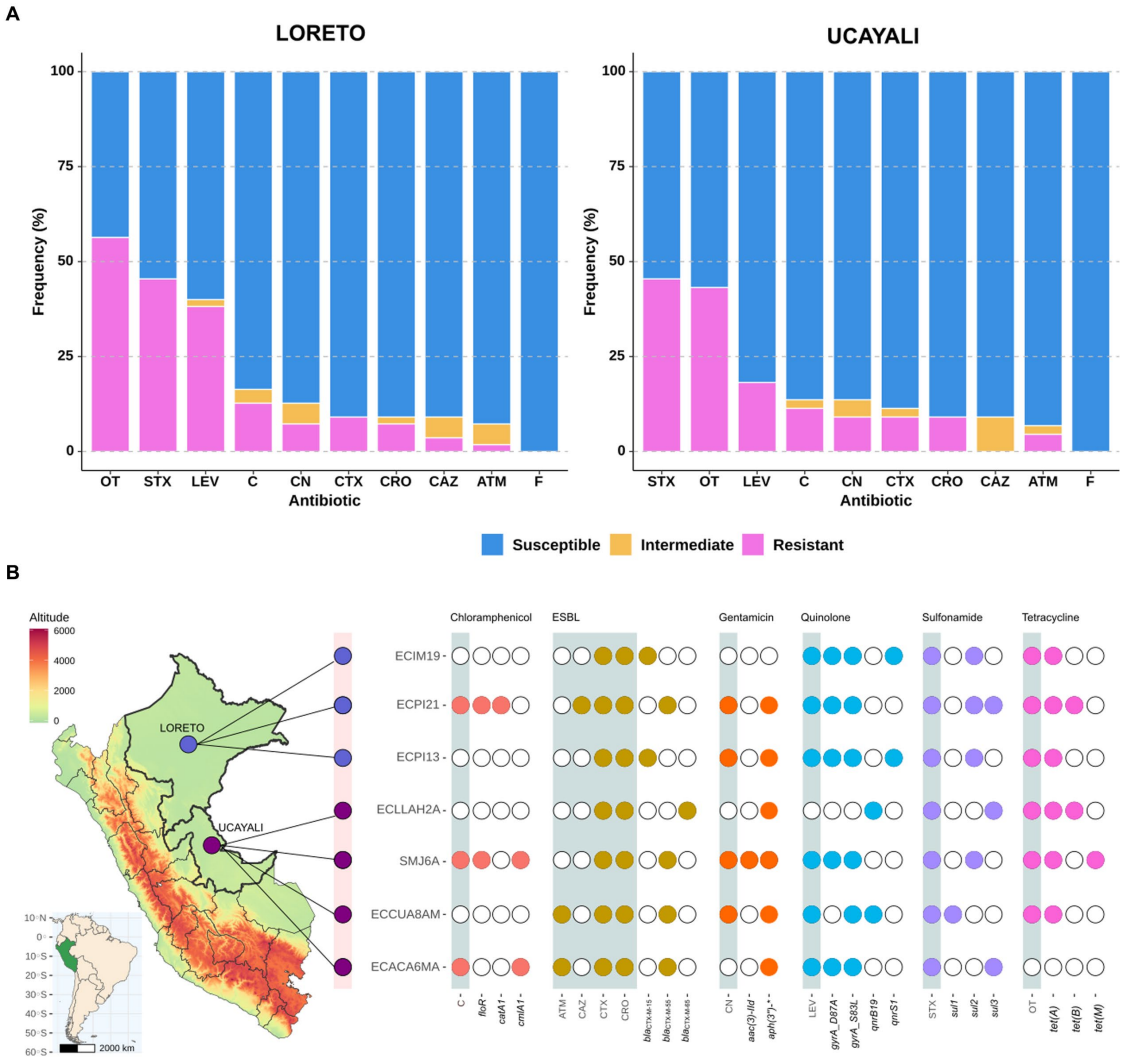
**(A)** AMR profile of *Escherichia coli* strains isolated from NHP in Loreto (*n* = 55) and Ucayali (*n* = 44) regions of the northeastern Peruvian Amazon; ATM, Aztreonam; C, Chloramphenicol; CAZ, Ceftazidime; CRO, Ceftriaxone; CTX, Cefotaxime; GEN, Gentamicin; LEV, Levofloxacin; STX, Trimethoprim-sulfamethoxazole; OT, Oxytetracycline; and F, Nitrofurantoin. **(B)** Identification map of Loreto (light purple) and Ucayali (dark purple) regions coupled to a diagram showing AMR phenotype distribution (gray shades) associated with the presence (color points)/absence (white points) of ARGs in seven genomes of ESBL-producing *E. coli* strains isolated from NHP.

Among 119 fecal samples obtained from NHP in two regions of the Peruvian Amazon, we isolated 99 *E. coli* strains. Of these, 55 strains were from semi-captive NHP in Loreto, and 44 were from captive NHP in Ucayali. Antimicrobial susceptibility tests revealed a similar resistance profile in samples from both regions. Approximately 45% of strains from both Loreto and Ucayali exhibited resistance to oxytetracycline and trimethoprim-sulfamethoxazole. Similarly, the proportion of strains resistant to chloramphenicol and gentamicin was below 10%. Additionally, 7.07% (7/99) of all strains—around 5.45% (3/55) from Loreto and 9.09% (4/44) from Ucayali—were identified as ESBL-producing *E. coli* (Figure 1A).

Whole genome sequencing was performed on the seven ESBL-producing *E. coli* strains to investigate ARGs (Figure 1B). We identified three variants of *bla*_CTM-X_: *bla*_CTX-M-15_ (two strains), *bla*_CTX-M-55_ (four strains), and *bla*_CTX-M-65_ (one strain). A broad resistome was also detected, conferring resistance to chloramphenicol (*floR*, *catA1*, and *cmlA1*), aminoglycosides (*aac(3″)-IId*, *aadA, aph(3″)-IIa*, *aph(6″)-Id*), quinolones (*qnrB19*, *qnrS1*), sulfonamides (*sul1*, *sul2*, and *sul3*), and tetracyclines (*tetA*, *tetB*, and *tetM*). Point mutations on the *gyr* gene (*gyrA_S83L* and *gyrA_D87A*) associated with quinolones resistance were also identified (Figure 1B). Additionally, genes encoding resistance against fosfomycin (*fos3A*) and lincomycin (*lnuG*), as well as a mutation in the *nfsA* gene (*nfsA_R15C*) associated with nitrofurantoin resistance, were detected (Supplementary Figure 1).

Among our seven ESBL-producing *E. coli* strains, five sequence types (ST) were identified: ST10, ST117, ST752, ST7176, and ST12254 (Table 1). Three of these strains (ECIM19, ECPI13, and SMJ6A) belong to the ST10 sequence type, a high-risk pandemic lineage previously reported in other hosts from South America. Additional genomic characteristics are detailed in Table 1 and Supplementary Table 1.

**Table 1:** Metadata summary of the seven ESBL-producing *Escherichia coli* genomes isolated from semi-captive and captive NHP in Loreto and Ucayali regions of the Peruvian Amazon.

Isolate	Host	Region	AMR profile	MLST	ST complex	HierCC HC50 scheme	O Antigen	H Antigen	Clermont Type
ECIM19	*Saguinus labiatus*	Loreto	CTX, CRO, OT, LEV, STX	ST10	ST10 Cplx	37	O9	H9	A
ECPI13	*Saguinus mystax*	Loreto	CTX, CRO, OT, CN, LEV, STX	ST10	ST10 Cplx	37	O9	H9	A
ECPI21	*Saguinus mystax*	Loreto	CAZ, CTX, CRO, OT, LEV, CN, STX, C	ST12254	ST10 Cplx	29,176	O89	H4	A
ECACA6MA	*Ateles chamek*	Ucayali	ATM, CTX, CRO, LEV, STX, C	ST117	-	31,974	O45	H4	F/G^*^
ECCUA8AM	*Cebus unicolor*	Ucayali	ATM, CTX, CRO, OT, LEV, CN, STX	ST752	ST10 Cplx	242,851	-	H40	A
ECLLA2HA	*Lagothrix lagothricha*	Ucayali	CTX, CRO, OT, STX	ST7176	-	242,852	O162	H27	B1
SMJ6A	*Sapajus apella*	Ucayali	CTX, CRO, OT, LEV, CN, STX, C	ST10	ST10 Cplx	71,429	O89	H10	A

ATM, Aztreonam; C, Chloramphenicol; CAZ, Ceftazidime; CRO, Ceftriaxone; CTX, Cefotaxime; GEN, Gentamicin; LEV, Levofloxacin; STX, Trimethoprim-sulfamethoxazole; and OT, Oxytetracycline. ^*^Before phylogroup F, now update to phylogroup G.

### 3.2 Phylogenomic analysis of ESBL-producing *Escherichia coli* strains

**Figure 2:**
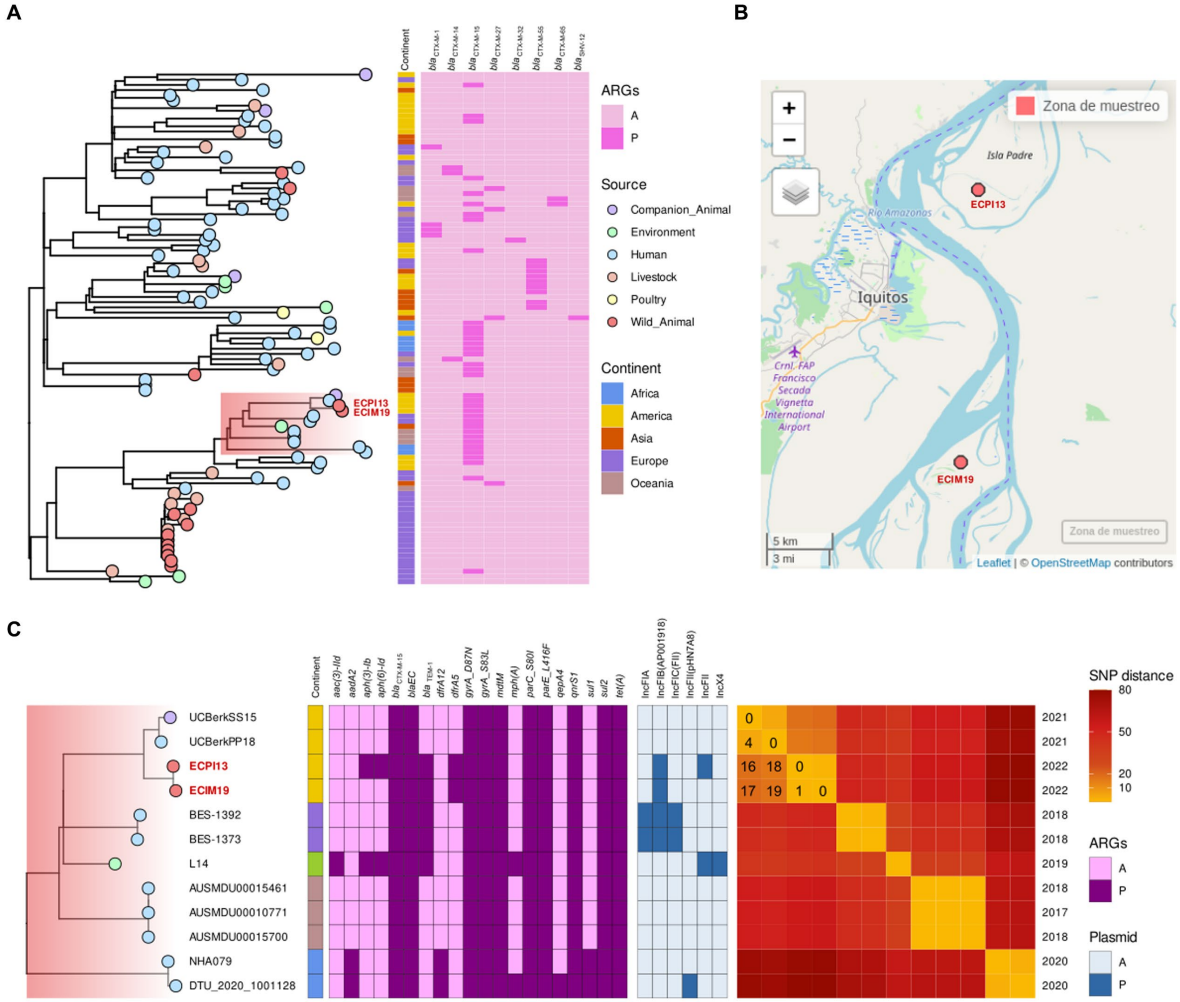
**(A)** Phylogenetic tree of *Escherichia coli* ST10 HierCC HC50_37 group downloaded from Enterobase, including ECIM19 and ECPI13 genomes (names in red), showing the source of strains (points), continent reported (squares), and presence (dark pink square)/absence (light pink square) of of *bla*_CTX-M_ variants. **(B)** Map of the sampling locations of ECIM19 and ECPI13 isolates in the Loreto region. **(C)** Subclade highlighted in “A,” including ECIM19 and ECPI13 genomes with closely related genomes, coupled to binary heatmaps, indicating the presence/absence of ARGs and plasmid replicon types, and an SNPs distance heatmap, specifying the divergent SNPs number and the isolation year.

Phylogenomic analysis of the HC50_37 cluster, which includes the ECIM19 strain from *S. labiatus* and the ECPI13 strain from *S. mystax*, revealed a SNP distance ranging from 0 to 519 SNPs between strains within this cluster. The majority of strains (*n* = 58/99) in this cluster harbored *bla*_CTX-M_ genes, indicating the presence of a high-risk CTX-M-producing *E. coli* sublineage with a broad host and geographic distribution (Figure 2A). Interestingly, the genomes of the ECIM19 and ECPI13 strains were remarkably similar, differing by only one SNP in the core genome alignment. This close genetic proximity suggests that both strains belong to the same transmission cluster, despite being recovered from two different monkey species (*S. labiatus* and *S. mystax*, respectively) on islands located 26.5 km away from each other (Figure 2B). Differences in genetic content between both strains rule out possible laboratory contamination; the ECIM19 strain differentially presents an IncFII plasmid and *aph(3)-Ib* and *aph(6)-Id* resistance genes that ECM13 strain lacks (Figure 2C).

Furthermore, within the subclade (highlighted in red) that includes monkey strains, there are two additional closely related strains: one from a human and one from a dog isolated in Quito, Ecuador, in 2021 (33). The SNP distance between our strains and Ecuadorian strains ranged from 16 to 19 SNPs, respectively, (Figure 2C). All members of this subclade contained the *bla*_CTX-M-15_ gene and other ARGs such as *qnrS1*, *sul2*, and *tet(A)*. These data provided compelling evidence that a CTM-M-15-producing *E. coli* clonal strain is widely disseminated among different hosts, including NHP and humans, in geographically separated locations from South America.

Phylogenetic analysis of the SMJ6A strain from *S. apella* and 19 genomes from HC50_71429 cluster shows that the SMJ6A strain was the most related to FP209CP strain from a pigeon (*Columba livia*) recovered in Oceania, with a distance of 506 SNPs (Supplementary Figure 2A). Similar results were obtained for the HC50_29176 cluster, where the ECPI21 was closely related to Ecuadorian strains from domestic dogs and swine with SNP distance of 94 and 96 SNPs, respectively, and shared the *bla*_CTX-M-55_ gene (Supplementary Figure 2B).

Finally, the phylogenetic analysis of the ST7176 lineage revealed that the ECLLAH2A strain from *L. lagothricha* was related to two poultry strains from the United States (Supplementary Figure 2C). Unlike the other strains, ECLLAH2A strain was the only one carrying the *bla*_CTX-M-65_ gene, while the other strains in the cluster showed the *bla*_CTX-M-55_ variant.

### 3.3 Genetic context of *bla*_CTX-M_ genes

**Figure 3:**
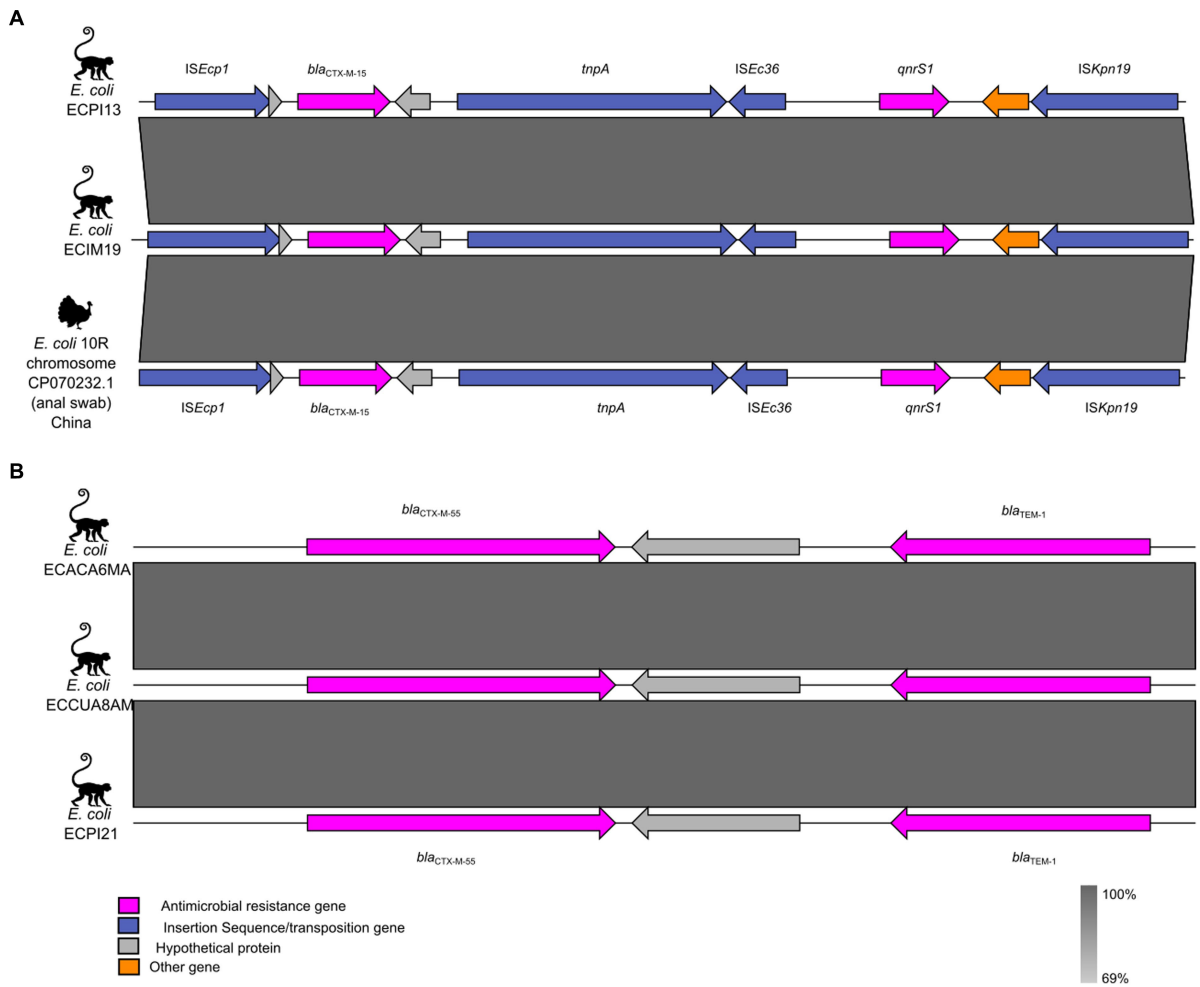
Genetic context of the *bla*_CTX-M_ variants in ESBL-producing *Escherichia coli* from NHP. **(A)** Genetic environment of *bla*_CTX-M-15_ and its neighboring mobile genetic elements. **(B)** Genetic environment of *bla*_CTX-M-55_. The ARGs, insertion sequences, and hypothetical proteins are represented by pink, blue, and gray arrows, respectively.

The genetic context of the *bla*_CTX-M_ genes is illustrated in Figure 3. The ECIM19 and ECPI13 strains carrying the *bla*_CTX-M-15_ variant were associated with the *ISEcp1* insertion sequence, identical to the one found in the chromosome of the *E. coli* 10R strain obtained from a turkey cloacal swab in China (Figure 3A). Additionally, the ECACA6MA, ECCUA8AM, ECPI21, and SMJ6A strains showed the presence of the *bla*_TEM-1_ gene adjacent to the ESBL-coding gene *bla*_CTX-M-55_ (Figure 3B). Unfortunately, due to the limitations of short-read sequencing, we could not assemble full plasmid sequences and explore the presence of mobile genetic elements surrounding the *bla*_CTX-M-55_ and *bla*_CTX-M-65_ variants in our strains.

### 3.4 Detection of virulent and hypermucoviscous ESBL-producing *Escherichia coli* strains

**Figure 4:**
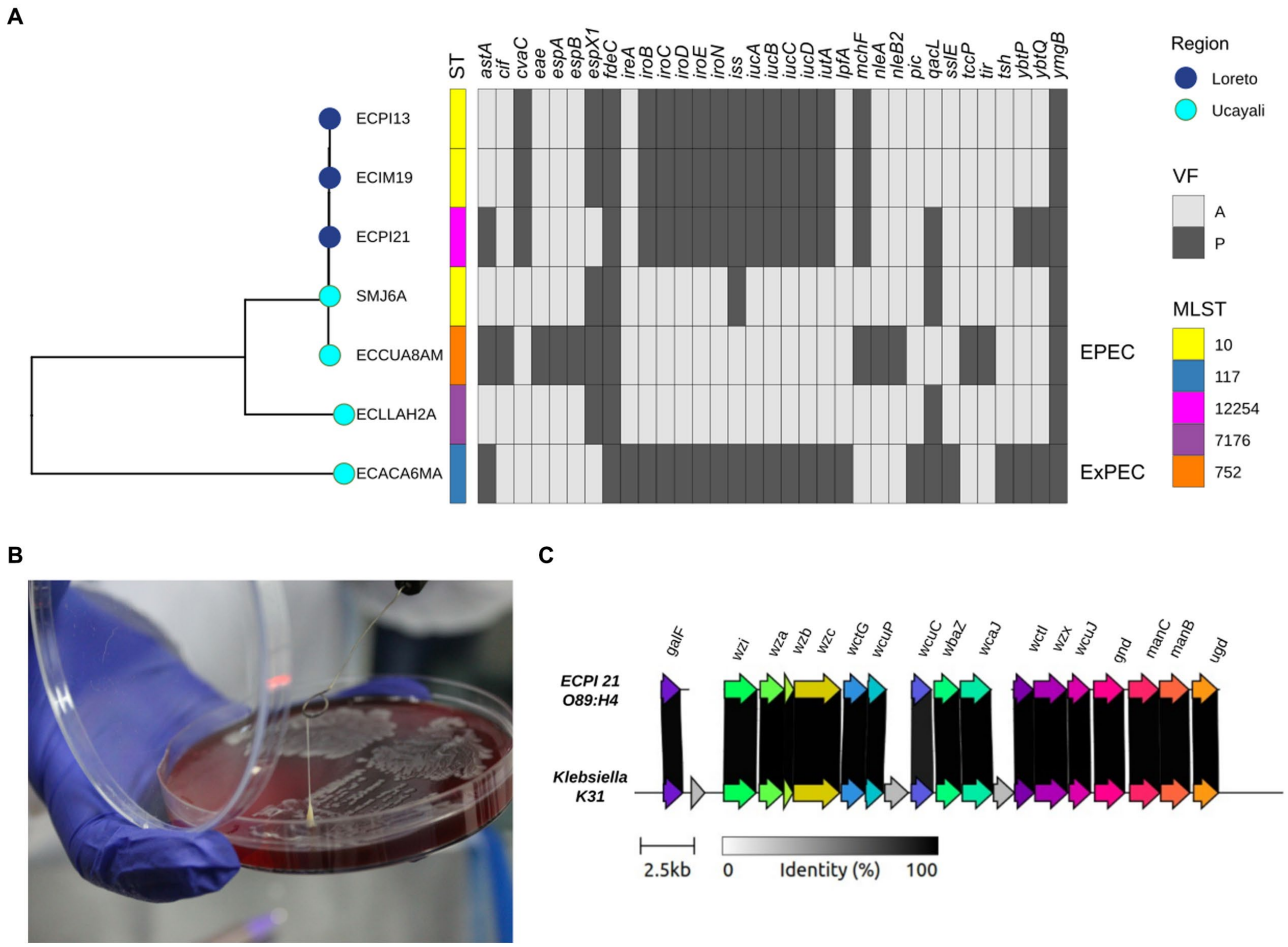
**(A)** Phylogenetic tree of seven ESBL-producing *Escherichia coli* strains from NHP coupled to a binary heatmap of presence (dark gray)/absence (light gray) of virulence genes (*n* = 33). **(B)** HMV phenotype of the ECPI21 strain isolated from *Saguinus mystax* represented by a positive “string test” (> 6 cm). **(C)** Capsule locus structure of HMV phenotype ECPI21 strain (O89:H4). The gray shading indicates the level of identity between ECPI21 and *Klebsiella* K31 reference capsule locus, which ranges from 98.10 to 100% nucleotide identity.

We identified 33 virulence genes in all seven ESBL-producing *E. coli* strains (Figure 4A). The genome of the ECCUA8AM strain from *C. unicolor* harbored the locus of enterocyte effacement (LEE) pathogenic island, containing *eae*, *tir*, *espA*, and *espB* genes that are associated with the Enteropathogenic *E. coli* (EPEC) pathotype (34). Additionally, we detected *ireA*, *iroN*, *iss*, *iutA*, *astA*, and *tsh* genes in the genome of ECACA6MA isolated from *A. chamek*, genes previously associated with Extraintestinal Pathogenic *E. coli* (ExPEC) pathotype in NHP (35).

The ECPI21 strain from *S. mystax* exhibited a hypermucoviscous (HMV) phenotype, as evidenced by the “string” effect of approximately 6 cm when adherent to a microbiological loop (Figure 4B). This characteristic resembles the HMV phenotype observed in certain virulent *Klebsiella pneumoniae* strains, providing an advantage for invasive infections (16, 36). While the identification of HMV strains in *E. coli* is uncommon, it has been described in livestock and clinical strains (37, 38). Analysis of the ECPI21 genome identified a highly conserved *Klebsiella*-capsule type K31 (98.86% identity; Figure 4C).

## 4 Discussion

We report the detection and genomic analysis of seven ESBL-producing *E. coli* strains in semi-captive and captive NHP from two regions of the Peruvian Amazon. ESBL-producing *Enterobacteriaceae* are critical priority resistant pathogens and were not previously reported in NHP from South America. A recent study found MDR-*Enterobacteriaceae* in samples taken from free-living black capuchin monkeys (*Sapajus nigritus*) in Brazil, identifying resistant strains to beta-lactam antibiotics, but no ESBL-production was detected (39). Our ESBL-producing *E. coli* strains were found to have broad resistome to relevant antibiotics (Table 1). These findings are consistent with previous reports of MDR and ESBL-producing *Enterobacteriaceae* isolated from fish, soils and aquatic environments in the Amazon ecosystems (40–42).

The most prevalent lineage in our dataset was the ST10 clone, a well-known high-risk pandemic lineage associated with infections in humans and has been found in livestock and meat in Peru (43, 44) Other STs identified in this study, such as ST117, have been found in emerging ExPEC strains of foodborne *E. coli*, posing a risk to human health (45, 46). ST752 has been associated with poultry in Europe and the United States and is considered predominant in chicken populations (47, 48). ST7176 has been detected in porcine *E. coli* strains carrying the *bla*_CTX-M-55_ variant (49).

The CTX-M family is the most widespread and clinically relevant ESBL enzyme (7). We identified three *bla*_CTX-M_ variants in our ESBL strains, with *bla*_CTX-M-55_ gene being the most common. Interestingly, recent surveillance studies have reported that this variant is also predominant in *E. coli* strains from pigs, cows, and chickens in Lima-Peru (43, 44, 50), suggesting that anthropogenic activities may serve as potential drivers of CTX-M-producing *E. coli* strains into the Amazonian wildlife. Conversely, *bla*_CTX-M-15_ and *bla*_CTX-M-65_ variants were found to a lesser extent in CTX-M-producing *E. coli* strains from livestock and bats in Peru (43, 50).

Strikingly, we identified two CTX-M-15-producing *E. coli* ST10 differed from each other by only one SNP across the entire recombination-free core genome. This genetic similarity is noteworthy, as both strains were sampled from two different monkey species, *S. mystax* and *S. labiatus*, located 26.5 km apart. The phylogenetic analysis also revealed that two CTX-M-15-producing *E. coli* from Quito, Ecuador (33), were, on average, 18 SNP distant from our strains. Several studies have demonstrated clonal expansion as a key mechanism for understanding the spread of MDR *E. coli* in diverse hosts and environments. The evidence suggests that specific MDR and CTX-M-producing *E. coli* lineages can spread among subjects geographically separated over an extended period with minimal variation in the core genome (51–53).

The remarkable genome similarity of CTX-M-15-producing *E. coli* ST10 supports the idea of clonal expansion of this lineage among semi-captive monkey populations in the Peruvian Amazon rainforest and other hosts in South America. The source of this clonal spread in the primate population remains unknown, with the most plausible explanation being transmission from humans to NHP populations. Due to the expansion of human settlements in natural NHP habitats in Loreto (Muyuy and Padre islands), the interaction between both hosts has become frequent, increasing the probability of pathogen transmission. Alternatively, there is growing evidence that antimicrobial resistance has impacted Amazonian soils and aquatic ecosystems (40, 42, 54); these environments could serve as reservoirs for AMR that wildlife such as NHP may acquire.

Two ESBL-producing *E. coli* strains isolated in captive NHP from the Ucayali carried virulence factors typically associated with the EPEC and ExPEC pathotypes. The EPEC pathotype is characterized by its ability to cause attaching-effacing (A/E) lesions, leading to diarrhea and dysentery in humans and livestock (55). We found genes associated with the locus of enterocyte effacement (LEE) pathogenic island in the ECCUA8AM strain isolated from *C. unicolor*. Previously, the EPEC pathotype was identified in captive young *Aotus* sp. specimens in Loreto, exhibiting diarrhea in some cases and apparently healthy in others, supporting the hypothesis of NHP as an important reservoir of pathogenic *E. coli* (56).

On the other hand, ExPEC is known to cause extraintestinal diseases in humans and has been isolated from NHP, although it remains unclear whether it causes disease in NHP (35). The ECACA6MA strain isolated from *A. chamek*, an endangered species according to the IUCN (57), contains genes associated with the ExPEC pathotype. Despite being pathogenic forms of *E. coli*, these strains were obtained from apparently healthy animals, suggesting that these NHP could act as reservoirs for those *E. coli* pathotypes and negatively impact the health of naïve NHP and Amazonian human populations.

The HMV phenotype observed in the ECPI21 strain from *S. mystax* may be associated with the conserved *Klebsiella*-K31 capsular sequence in its genome, since K31 capsular type has been linked to HMV *K. pneumoniae* in various hosts (16, 58, 59). Functional assays are necessary to confirm the association between K31 capsule type and the HMV phenotype observed in ECPI21. The coexistence of ESBL and HMV phenotypes should be a significant concern for public health due to their potentially enhanced virulence (16, 36–38).

In conclusion, we report the isolation of MDR and ESBL-producing *E. coli* from semi-captive and captive NHP in two regions of the Peruvian Amazon. Genomic analysis revealed three different *bla*_CTX-M_ gene variants (*bla*_CTX-M-15_, *bla*_CTX-M-55_, and *bla*_CTX-M-65_) and a wide resistome conferring resistance to relevant antibiotics. Furthermore, two strains were characterized as EPEC and ExPEC pathotypes according to their virulence factors, and one more presented HMV phenotype. Most ESBL-producing *E. coli* strains were assigned to the high-risk pandemic ST10 sequence type, and two of these were closely relatedness to high-risk pandemic lineages previously reported in humans and domestic animals.

This diversity suggests potential environmental pollution resulting from human activities associated with the use of antimicrobial compounds. The presence of pathogenic strains carrying a broad resistome in NHP facilitates the persistence and rapid spread of critical priority ESBL-producing *E. coli*, which may have a negative impact on the conservation of the Amazonian wildlife and their natural environments. This underscores the importance of adopting the One Health approach in the AMR surveillance, with the aim of minimizing the potential risk of transmission of antibiotic-resistant bacteria and anticipating the emergence and spread of zoonotic and anthroponotic diseases at the human-NHP interface.

## Data availability statement

The original contributions presented in the study are publicly available. This data can be found at: https://www.ncbi.nlm.nih.gov/; PRJNA992559.

## Ethics statement

The animal study was approved by Comité de Ética y Bienestar Animal—CEBA—UNMSM (Authorization No. 2022-10) and Servicio Nacional Forestal y de Fauna Silvestre (SERFOR) with Directorate Resolution No. RD-000031-2022-MIDAGRI-SERFOR-DGGSPFFS-DGSPFS. The study was conducted in accordance with the local legislation and institutional requirements.

## Author contributions

JB-G: Conceptualization, Formal Analysis, Methodology, Writing – original draft, Writing – review & editing. TS-E: Formal analysis, Methodology, Writing – review & editing. CR: Formal analysis, Methodology, Writing – review & editing. DC: Formal analysis, Methodology, Writing – review & editing. YI: Formal analysis, Methodology, Writing – review & editing. LL: Conceptualization, Formal analysis, Funding acquisition, Writing – original draft, Writing – review & editing. RR: Supervision, Writing – review & editing. LM: Conceptualization, Formal analysis, Funding acquisition, Supervision, Writing – original draft, Writing – review & editing.

## Footnotes

^1^ https://github.com/tseemann/snippy
