# Supplementary material for: Genomic insights into ESBL-producing Escherichia coli isolated from non-human primates in the Peruvian Amazon
Source: Front Vet Sci. 2024 Jan 16;10:1340428. doi: 10.3389/fvets.2023.1340428 (PMC10825005; doi:10.3389/fvets.2023.1340428)
Supplement: Supplementary file 1 [file Data_Sheet_1.pdf]

Supplementary Table 1: Quality parameters assembly of the seven ESBL-producing *E. coli* genomes isolated from semi-captive and captive NHP in Loreto and Ucayali regions of the Peruvian Amazon.

**Strain**	**Host**	**Region**	**Coverage**	**N50**	**Genome Size**	**%CG**
ECIM19	*Saguinus labiatus*	Loreto	121.9	130967	4796816	50.78
ECPI13	*Saguinus mystax*	Loreto	119.1	172736	4785694	50.8
ECPI21	*Saguinus mystax*	Loreto	129.9	104770	5043680	50.73
ECACA6MA	*Ateles chamek*	Ucayali	181.4	69418	5198309	50.71
ECCUA8AM	*Cebus unicolor*	Ucayali	57.4	42990	5398490	50.5
ECLLA2H	*Lagothrix lagothricha*	Ucayali	123.5	99792	4825434	50.62
SMJ6A	*Sapajus apella*	Ucayali	283.8	122436	4665599	50.84

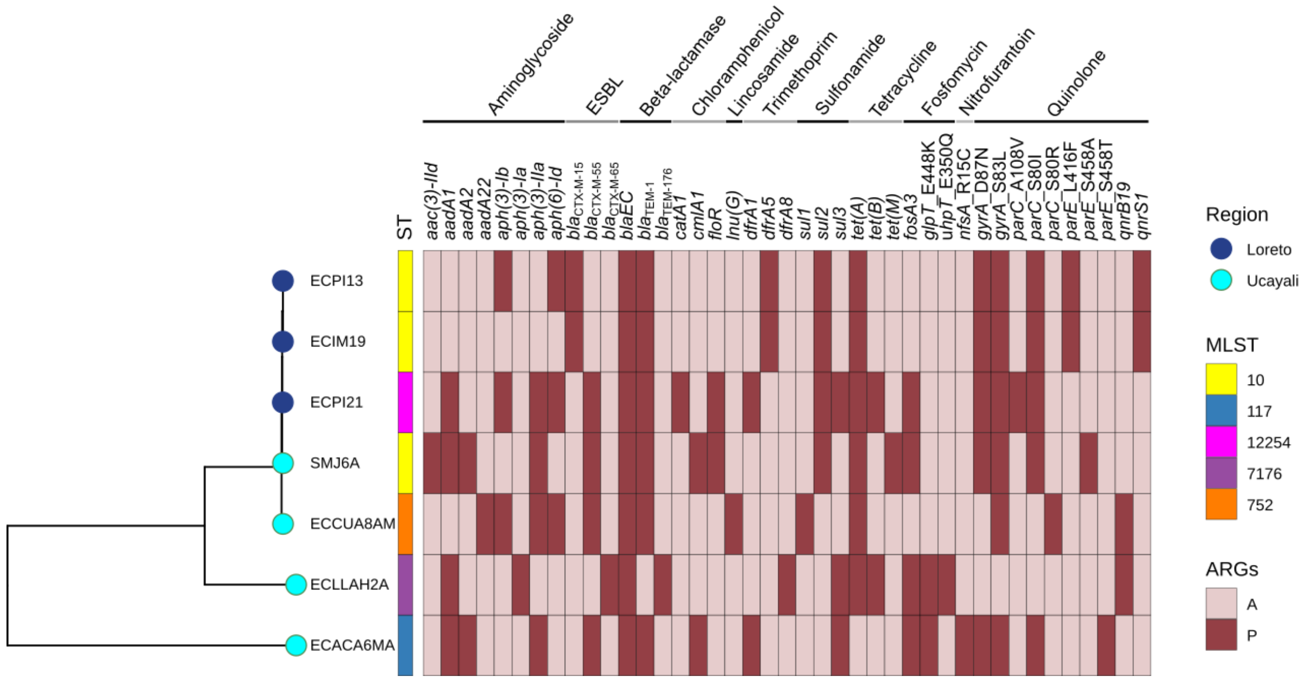

Supplementary Figure 1. SNP-based phylogenomic tree of seven ESBL-producing *E. coli* genomes isolated from NHPs from Loreto and Ucayali, annotated with their MLST genotype, and a binary heatmap of presence (dark red)/ absence (pink) of ARGs (n=30) and point mutation (n = 11) conferring resistance.

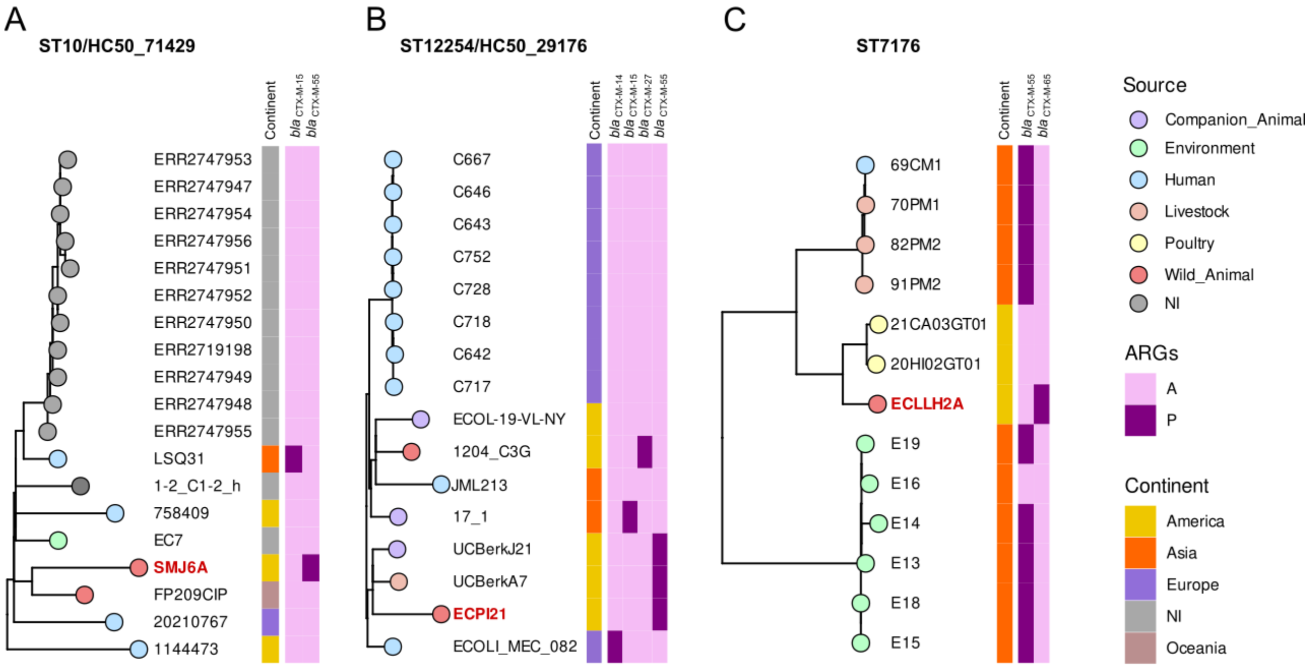

Supplementary Figure 2. (A) Phylogenetic tree of 18 genomes of the HierCC HC50_71429 cluster that were downloaded from Enterobase, including SMJ6 strain isolated from *S. apella*. (B) Phylogenetic tree of 15 genomes of the HierCC HC50_29176 cluster including ECPI21 strain isolated from *S. mystax*. (C), Phylogenetic tree of 12 ST7176 genomes including ECLLH2A strain isolated from *L. lagothricha*. All trees are annotated with their strain source, continent of strain, and presence (dark purple)/ absence (light purple) of $bla_{\text{CTX-M}}$ variant. NI: Not informed.
